# The Head-fixed Behaving Rat—Procedures and Pitfalls

**DOI:** 10.3109/08990220.2010.513111

**Published:** 2010-10-18

**Authors:** Cornelius Schwarz, Harald Hentschke, Sergejus Butovas, Florent Haiss, Maik C Stüttgen, Todor V Gerdjikov, Caroline G Bergner, Christian Waiblinger

**Affiliations:** 1 Systems Neurophysiology Group, Werner Reichardt Centre for Integrative Neuroscience, University of Tübingen Germany; 2 Department for Cognitive Neurology, Hertie Institute for Clinical Brain Research, University of Tübingen, Germany; 3 Section for Experimental Anesthesiology, University of Tubingen, Germany; 4 School of Psychology, University of Leicester, Leicester, UK

**Keywords:** head fixation, psychophysics, operant conditioning, whisker, rodent

## Abstract

This paper describes experimental techniques with head-fixed, operantly conditioned rodents that allow the control of stimulus presentation and tracking of motor output at hitherto unprecedented levels of spatio-temporal precision. Experimental procedures for the surgery and behavioral training are presented. We place particular emphasis on potential pitfalls using these procedures in order to assist investigators who intend to engage in this type of experiment. We argue that head-fixed rodent models, by allowing the combination of methodologies from molecular manipulations, intracellular electrophysiology, and imaging to behavioral measurements, will be instrumental in combining insights into the functional neuronal organization at different levels of observation. Provided viable behavioral methods are implemented, model systems based on rodents will be complementary to current primate models—the latter providing highest comparability with the human brain, while the former offer hugely advanced methodologies on the lower levels of organization, for example, genetic alterations, intracellular electrophysiology, and imaging.

## Introduction

Rodents, mainly mice and rats, are the most frequently employed model organisms for the study of mammalian neuronal function and behavior. They are characterized by low breeding and housing costs, high reproduction rate, and ease of handling, combined with an enormous learning capacity and a rich behavioral repertoire. The anatomy of rodent brains is well known, and the lisencephalic layout of neocortex (i.e., the absence of any noteworthy gyri or sulci) is of general advantage for the study of neocortical function because it allows direct unobstructed access to somatotopic brain maps on the surface of the neocortex. The advent of genetically engineered mice was a breakthrough that allowed a multitude of approaches for the study of the function of molecules and their consequences on subsequent hierarchical levels of neuronal function.

Despite the power of rodent models in the investigation of the brain at multiple levels, in the study of sensorimotor, perceptual, and cognitive functions, the “awake behaving monkey” preparation is leading. Besides the obvious advantage of higher similarity of monkey and human brains, one important achievement of the awake monkey preparation is the ability to combine head-immobilization with the training of complex behavior. This allows superior precision in both stimulus application and behavioral assessment. For instance, receptive fields of sensory neurons can be stimulated in precise ways to assess perception (e.g., Britten et al. 1992), and motor output of hand and eye can be quantified at high precision (e.g., Fuchs 1967; Evarts 1968b; Wurtz 1969; Georgopoulos et al. 1982). While the monkey preparation connects neurobiological models to the human brain and is therefore of utmost importance, we believe that a viable “head-fixed behaving rodent” preparation is still desirable. At this point we want to emphasize the distinction between “head-fixed behaving” and “freely running” rodent preparations. The latter approaches are well established and study trained or spontaneous behaviors that rodents are able to perform in very small places. These methods will continue to be important for the study of sophisticated sensorimotor and cognitive behaviors in their largely native form, without unnatural physical restriction and without the necessity of over-training animals on a given task. In this paper, however, we will focus on novel approaches to head-fixate rodents which hold the promise that investigation of cellular, subcellular, and molecular processes can be related to sensorimotor, perceptual, and cognitive processes on a precise spatio-temporal scale. Besides the mentioned advantages of rodent model systems, the sheer wealth of existing genetic lines (certainly in mice but also in rats) is an asset that is not offered by any other mammalian animal model. By now, a multitude of proteins have been studied using genetically engineered mice, leading to an unprecedented knowledge on the effects of single molecules on more complex neuronal function, behavior, and disease. On the cellular level, intracellular electro-physiology has been used widely in rodent neuronal tissue *in vitro* but also *in vivo* during the last two decades. While intracellular recordings in anesthetized cats was one of the early advances to understand the function of the intact brain (Brock et al. 1952), nowadays a decisive role is played by the whole cell patch clamp technique (Neher and Sakmann 1976) which recently has been adapted to allow the first intracellular recordings from neocortex in awake behaving mice (Margrie et al. 2002; Crochet and Petersen 2006). In contrast, intracellular recording in awake behaving monkeys has never taken off, supposedly due to technical difficulties and reasons of inefficiency. Imaging techniques are yet another approach to gain insight in cellular mechanisms that show clear advantages if applied in rodents. While intrinsic imaging and voltage-sensitive dye techniques have successfully been used in monkeys (Grinvald and Hildesheim 2004), their routine application nowadays is in rodents. Recently, the first voltage-sensitive dye images of neocortical activity have been made available from spontaneously behaving mice using glass fiber optics (Ferezou et al. 2006, 2007). Another promising visualization of cellular function is two-photon-based calcium imaging which readily allows monitoring of neuronal activity in layers 2/3 of neocortex in rats and down to layer 4 in mice (Kerr and Denk 2008). Monkeys' strong meninges, sulci and gyri, and extended cortical depth (>2 mm, as compared to ∼1.5 mm in rats and ∼1 mm in mice) are hurdles for the successful application of two-photon imaging *in vivo* that will not easily be overcome. In the meantime the first imaging experiments have been successfully performed in awake, quiescent rats (Greenberg et al. 2008) and mice running on a spherical treadmill (Dombeck et al. 2007).

These advantages call for a head-fixed behaving rodent preparation that would establish a missing link to neurobiological research in monkeys: while research on awake monkeys is indispensable for investigations of human-like brain capabilities, the “head-fixed behaving rodent” is sufficient or, as will be argued below, in some cases even better suited to the analysis of cellular, subcellular, and molecular processes and their relationship to sensorimotor and/or cognitive processes.

## State of the art

Rodent head-fixation has been employed for many years, although the numbers of publications appear meager compared to the wealth of work based on the behaving monkey. Physiological processes without a behavioral observation or task (Sapienza et al. 1981), cardiovascular function (Parry and McElligott 1993), anesthesia effects (van Looij et al. 2004; Hentschke et al. 2005), facial function (Hadlock et al. 2007), and neuroimaging (Dombeck et al. 2007) have been investigated. The method has been further extended to the study of spontaneous eye movements (Sakatani and Isa 2007) and classical conditioning or reflex adaptation: eye blink conditioning in mice and rats (Welsh 1998), nictitating membrane reflex in rabbits (Disterhoft et al. 1977), as well as vestibulo-ocular reflex adaptation in rabbits (Precht et al. 1976). Operant conditioning of movements in head-fixed rats was first established by Welsh et al. (1995) who investigated licking movements, followed by other investigators who implemented conditioning of whisker movements (Bermejo et al. 1996, 1998; Hentschke et al. 2006) and lever presses (Harvey et al. 2001; Isomura et al. 2009). We have used precise whisker deflection together with conditioned licking movement as a simple instrumental response that indicates the decision of the animals in a psychophysical task and at the same time leads to reward consumption (Stüttgen et al. 2006; Stüttgen and Schwarz 2008, 2010; Gerdjikov et al. 2010). A paradigm that combines free whisking and psychophysical assessment in mice has been introduced recently (O'Connor et al. 2010).

The advantages of head-fixation as compared to the freely running rodent preparation are manifold. The head-fixed animal offers greater experimental control over sensory inputs and motor outputs. In the case of the rat whisker system, the presumed high tactile discrimination performance of rats (Carvell and Simons 1990) calls for precise whisker stimulation—“precise” implying micrometer precision on a sub-millisecond timescale. Recently, we reported the first behavioral assessment of whisker psychophysics in head-fixed rats, using stimuli that reached this level of precision (Stüttgen et al. 2006). Concerning motor output, we and others (Bermejo et al. 1998; Haiss and Schwarz 2005; Hentschke et al. 2006) demonstrated measurements of actively generated whisking trajectories with and without obstructing objects, again with micrometer and millisecond precision. Despite recent advances, the assessment of detailed movement trajectories in freely moving animals is complicated, requiring elaborate video-graphic tracking techniques (Ritt et al. 2008; Voigts et al. 2008). Even if animals are not trained on a particular task, the head-fixed awake preparation offers the advantage that electrophysiological signals are untinged by the effects of anesthesia on the one side, and less prone to contamination with artifacts originating from the animal's movements on the other. Furthermore, head-fixation provides favorable conditions of mechanical stability needed to visualize neuronal signals using calcium or voltage-sensitive dyes in awake animals (Dombeck et al. 2007; Ferezou et al. 2007; Greenberg et al. 2008).

The slow pace with which the head-fixed behaving rat is winning recognition in the field of systems and cognitive neurobiology is, to our understanding, first due to the extensive time needed to habituate the animal to head-fixation. The second, more nagging disadvantage of the rodent head-fixed preparation is that the behavioral repertoire of rodents includes many whole-body movements that are, by definition, impossible to perform under head-fixation. It is thus very important to devise a selection of head-fixation-compatible movements. Optimally, these would be movements that the animals use under natural conditions as well. This paper will therefore focus on the problem of adaption to head-fixation and will discuss natural movements like whisking, licking, and fore-paw movements (grasping and lever pressing) that are amenable to training in the head-fixed condition.

## Experimental procedures and pitfalls

### Animals

The choice of the strain and gender of the animal is an important consideration. In principle, however, the two strains we have worked with so far, Sprague Dawley (SD) as well as Long–Evans rats (LE), both inbred and delivered by Charles River (Sulzfeld, Germany), can be used for behavioral training under head-fixation. In our experience, LE are more agile and tend to explore more as compared to SD. Overall, the training of movements, especially whisker movements, seems to be a bit easier using LE. This advantage, however, is markedly dented by the tendency of LE to be more nervous and fearsome, and thus, to habituate much slower to the head-fixation procedure. Given the same duration of habituation the level of stress of LE under head-fixation is larger, and thus their overall performance tends to be worse than that of SD. An additional disadvantage of LE rats is the recent finding that many of them may be prone to fits of absence epilepsy (Shaw 2007)—the main reason why we have recently abandoned their use.

Concerning sex, we find that both male and female rats are amenable to operant conditioning under head-fixation. However, there are differences between the sexes that need consideration. Females have smaller body weight and are weaker, and they gain weight more slowly after the age of 12 weeks than males. These factors are of advantage for head-cap stability, since females are less forceful during fixation, exerting less mechanical stress on the head-cap. However, these advantages are offset by the stronger skull of males which allows a superior anchoring of the head-cap onto the bone using screws. Also, the smaller body weight of females results in less daily water intake, a factor which has to be considered when choosing the volume of a liquid reward (see below). In our hands, effects of the estrus cycle on the training of female rats have been detected only in sporadic cases.

The optimal age for implantation of a head-cap is 12 weeks (250–300 g body weight). Females are close to full-grown at this time while males can grow for a longer time. However, most of the total growth of both sexes is completed at this time (Weiler and Farbman 1997). Implantation of older rats is possible, but we found that old rats learn less well and are more difficult to habituate to the whole procedure.

Behaviorally trained animals are best kept under an inverted light schedule (12h dark 12h light, e.g., light on 8 p.m.) as training during daytime working hours thus coincides with the physiological activity period of rodents. With respect to water control it is important to realize that water intake in rodents is directly coupled to the animals' internal rhythm and is highest during the activity (dark) phase—independent of parameters of water homeostasis (Fitzsimons 1957). The room for behavioral training and handling should be dimly lit to interfere minimally with the animals' daily activity schedule.

It is advantageous to train littermate rats in parallel and house them together in one cage (we commonly house rats in pairs). This measure allows social interaction, important for the animals' well-being and the reduction of stress. With pair-wise housing one caveat is, however, that the animals should be separated after surgery for about 2 weeks to avoid the manipulation of fresh wounds by the cagemate. Another disadvantage is the risk of transmission of infections (which may occur at later stages).

### Pre-surgery handling

Pre-surgery handling should commence 2 weeks before surgery. It is essential to ensure the animal becomes comfortable with the experimenter and does not associate the experience of surgery with this person. Most important is the rat's acquaintance with the experimenter's voice, smell, and touch. Pre-surgery handling should begin with simply placing the hand in the cage and allowing the rats to explore it. This can be repeated multiple times for one or two days. Next the rat should be accustomed to being picked up. A mistake commonly made by new experimenters is to assume that this means taking the animal out of the cage and holding it in one's lap etc., for a lengthy period of time. This is not necessary and may even be counterproductive as it deprives the rat of the safety provided by its own cage at this early stage of “mutual introductions”. In fact, it is sufficient to lift the animal at one side of the cage and immediately release it at the other, repeating this simple movement a number of times (say 50). This should be extended by releasing the animal on the workbench and waiting 1–2 s before placing it back in the home cage. It is at this stage, rather than after surgery, that standard rodent treats can be introduced to reinforce the rats' natural curiosity and interest in the experimenter.

### Implantation of head-cap and maintenance

Oral antibiotics (Baytril; Bayer HealthCare, Leverkusen, Germany, 2.5% in 100 ml drinking water) are provided for 3 days before surgery and 1 week post-operatively. Animals are anesthetized using ketamine and xylazine (100mg and 15mg per kg body weight, respectively). The head fur is shaved and depilatory cream is used to remove the hair entirely. After depilation the cream should be swabbed and rinsed away completely, as the animals tend to ingest it after waking up due to enhanced grooming. Depilation is useful to provide a clean wound cleft. In addition, remaining hair tends to interfere with adhesion of the skin and provides a possible entrance path for pathogens. Throughout surgery, the eyes are covered with ointment to prevent drying out and infection of the cornea. If surgery is expected to last more than 3–4 h, glucose solution is injected subcutaneously (5 ml every 5 h of sterile 4% glucose in saline).

The rat is transferred to a stereotaxic device and mounted using blunt ear bars that do not break the eardrums. Body temperature is measured by a rectal probe and held constant at 37°C using a controlled pad. Anesthesia is continued at this point using isoflurane in oxygen. Concentration of isoflurane is adjusted (rat minimum alveolar concentration is ∼1.5% (Mazze et al. 1985)), such that pain reflexes are blocked (foot withdrawal after pinch with a forceps between the toes).

After disinfection the skin overlying the skull is incised using a scalpel and the soft tissue and muscles are removed from the surface of the skull. It is important to remove the temporal muscles partially on both sides of the head from the bone using a bone curette. This makes it possible to form the head-cap such that it embraces the skull from both sides, conferring extra stability. In order to prevent post-surgery problems with chewing, the removal of the temporal muscle should be limited to the first 2–3 mm below the lateral rim of the skull. Temporal muscle and skin are kept moist, and are gently extended away from the skull using surgical sutures attached to hemostats. At this time the skull is cleaned, disinfected (H_2_O_2_, 3%), rinsed, and then prepared for anchoring the head-cap by creating microcavities in the bone. To this end the skull is irrigated with phosphoric acid (GLUMA Etch 20 Gel; Heraeus Kulzer GmbH, Hanau, Germany, or Gel Etchant; Kerr Corporation, CA, USA) for 30 s. Etching with phosphoric acid is best done before any metal parts like screws or wires are attached to the bone to prevent unwanted chemical reactions. Care is taken during this procedure to prevent contact of phosphoric acid with soft tissue. After this, the phosphoric acid is carefully and thoroughly removed by a suction pipette, and the skull is rinsed with sterile saline.

**Figure 1:**
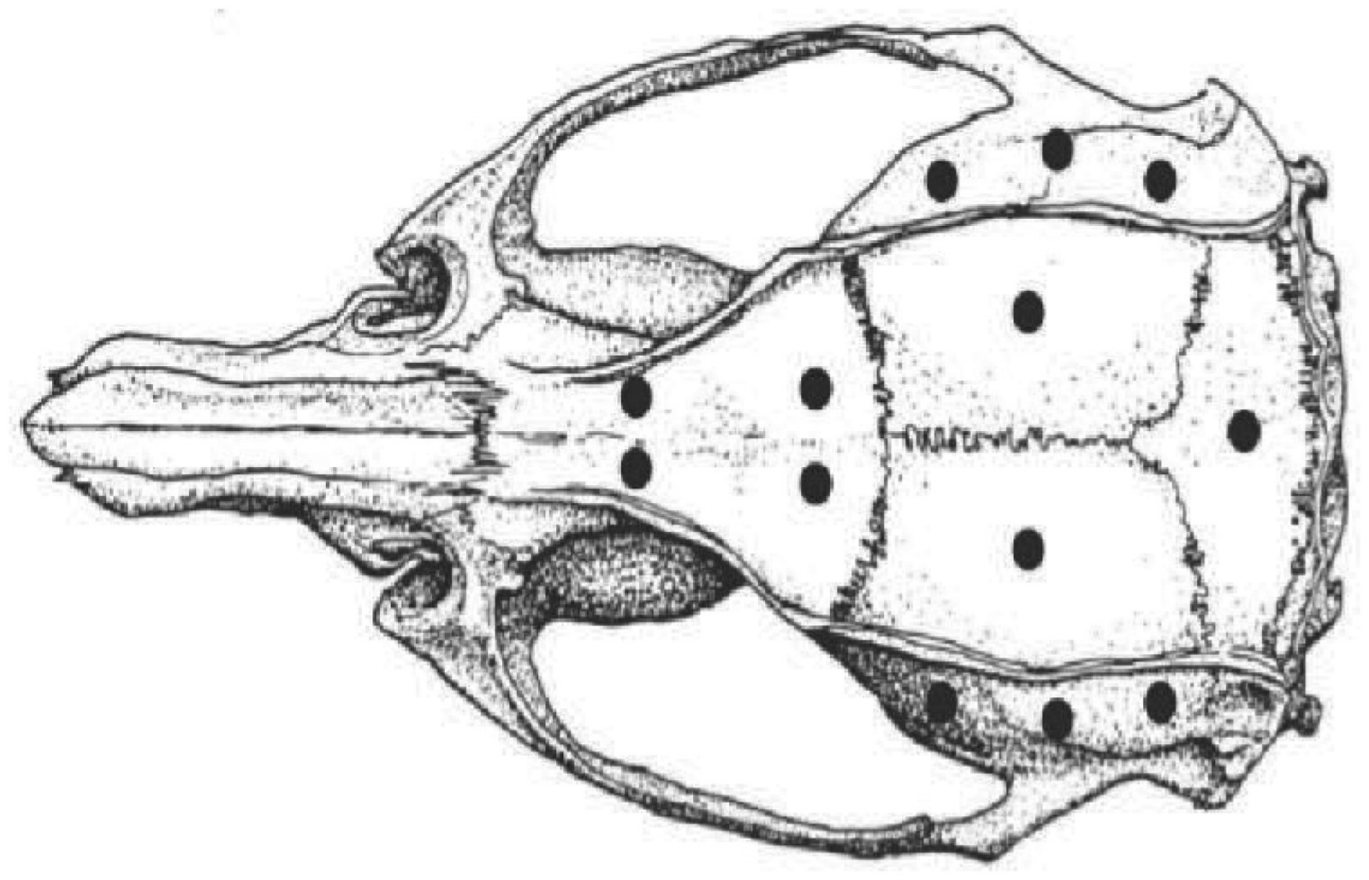
Head implant. Shown is a skull of a rat with positions of burr holes (0.007″) marking the position of skull screws. Modified from Paxinos and Watson (1986).

Burr holes of 0.7 mm diameter are then made at the locations shown in Figure 1. Self-tapping stainless steel screws (Small Parts, Logansport, IN, USA, part number TX0–2; or Morris Co., Southbridge, MA, USA, part number 0 × 1/8 flat), disinfected in ethanol 70–80% and dried for 30 s, are screwed into the pre-drilled holes. Maximally two turns should be applied to the screw, otherwise the tip of the screw will protrude into the head cavity and may potentially damage the surface of the brain. The sharp tips of the screws can be filed to a round shape to lessen the chance that the underlying brain tissue gets harmed. In any case, it is common sense to avoid placing skull screws above brain areas critical for the experiment at hand. In the screw map shown in Figure 1, individual screws can be omitted without any risk of instability. The screws on top of the olfactory bulb lend most stability to the head-cap as frontal bone tends to be very firm. Lateral skull bone is much softer and thinner. Nevertheless, the screws therein are also important because as mentioned, they provide a clamp of the skull from the two sides, providing extra stability. This is the appropriate time to drill additional holes through the skull for the placement of electrodes (e.g., silverball electrodes on the surface of the brain). After placing these electrodes the holes have to be closed using bone wax or dental cement.

The next step is the application of the bonding agent which mediates adhesion of the head-cap made of dental cement to the bone. Before doing this, remaining fluids (e.g., emerging from sutures) must be swabbed off, as they may compromise skull-to-cement bonding. The liquid bonding agent (Optibond FL; Kerr Corporation) is applied to the absolutely dry bone and cured using a light gun (Jovident International B.V., Eindhoven, the Netherlands). Immediately following this, a first thin layer of dental cement is applied (Tetric EvoFlow, Ivoclar Vivadent AG, Schaan, Liechtenstein).

The trepanation for the electrodes is then performed (if needed), the electrodes implanted, and the head-cap is built up according to experimental requirements. The simplest head post is a screw (thread 6 mm diameter for rats), to be embedded head-down into the dental cement. The best location for the head post, if compatible with the experimental constraints, is at the midline above lambda. Other locations are also possible, but extreme positions (i.e., craning over the rim of the skull etc.) should be avoided as they result in large or asymmetric torques on the skull screws when the animal activates its neck muscles. This one-point fixture is appropriate for extracellular electrophysiology using chronically implanted electrodes. For approaches that require maximum stability, for example, imaging or intracellular electrophysiology, a head plate (Greenberg et al. 2008) or a four-point fixture (Isomura et al. 2009) is advantageous. At the end of the surgery, before suturing, any sharp edges of dental cement are filed off, and the situs is disinfected and rinsed. This is the time to paint the surface of the dental cement with silver paint (needed for shielding in case electrophysiological recordings are planned, see next section). The temporal muscle is attached back to the skull, and the skin is sutured rostrally and caudally such that it attaches gently (but without gaps) to the dental cement. The wound is treated with antibiotics (Nebacetin; Astellas Pharma GmbH, München, Germany).

The rat is released from the stereotaxic and kept warm using infrared light. The first days post-surgery rats are kept on cellulose, which in contrast to standard bedding material will not adhere to wounds or eyes. Foot pellets are soaked in water to facilitate eating and water uptake. We inject 5 ml of glucose solution (s.c.) twice daily during the first few days after surgery until the animal feeds well by itself. As analgesic, caprofen (5 mg/kg, i.m.; Pfizer GmbH, Karlsruhe, Germany) or buprenorphine (0.05 mg/kg; Essex Pharma GmbH, München, Germany) is injected twice a day. Buprenorphine, a morphine derivate, is an excellent analgesic, but causes a number of side effects, for example, drowsiness, and has a potential for respiratory and digestive complications. In our experience these side effects are much stronger in LE compared to SD rats. Individual LE rats under buprenorphine were very weak and lethargic, lying on their side, and were not able to drink and eat for a prolonged period. Therefore, for LE rats at least, we recommend the use of caprofen which never caused these side effects. Rats and mice under caprofen are pain free and agile and commence to eat and drink very soon after surgery (often on the first day) and thus recover much faster. The antibiotic treatment started before the surgery is maintained for another week.

Properly recovered, the animal will clean the head-cap and the abutting skin by itself. If the skin surrounding the head-cap shows signs of inflammation it has to be cleaned and disinfected and treated with anti-inflammatory ointment (Octenivet Wound Gel; Schülke & Mayr GmbH, Norderstedt, Germany). If needed, oral antibiotic treatment can be reinstantiated for 7 days. Behavioral training can be continued after 2 weeks of recovery.

### Electrophysiology

Electrophysiology in our lab is based on pulled glass-coated platinum tungsten electrodes (80 μm shank diameter, 23 μm diameter of the metal core, free tip length <10 μm, impedance >1 MΩ; Thomas Recording, Giessen, Germany) that are placed inside an array of polyimide tubing (HV Technologies, Trenton, GA, USA), and are moveable by turns of a carrier screw (250 μm per revolution, Haiss et al. 2010). After each successful recording session, the electrodes can be lowered by a quarter to half a revolution. The electrodes are soldered to Teflon-insulated silver wires (Science Products, Hofheim, Germany), which in turn are connected to a micro-plug (Burklin, Munich, Germany). Parameters of the electrode arrays like inter-tip distance, electrode length, and geometric outline of the array (1 × 4 to 3 × 3, different extent of electrodes along their longitudinal axes, etc.) can be fashioned according to the structure to be recorded from and specific experimental requirements. With these microelectrodes simultaneous LFP and spike recordings can be performed using proper hardware (preamplifiers, filter, and amplifiers, MultiChannelSystems, Reutlingen, Germany; gain 5000, sampling rate 20 kHz). For the LFP signals we use a reference signal from the head screw above the cerebellum. The signal is filtered with a band pass at 1–200 Hz and stored on a PC. For spikes, a low impedance microelectrode (50.5 MΩ) within the array serves as reference to reduce high frequency noise stemming from muscle activity. The skull screw above the olfactory bulb is used as animal ground. The head-cap is covered with silver paint at the end of the surgery and grounded.

### Equipment to control behavior and whisker stimulation

**Figure 2:**
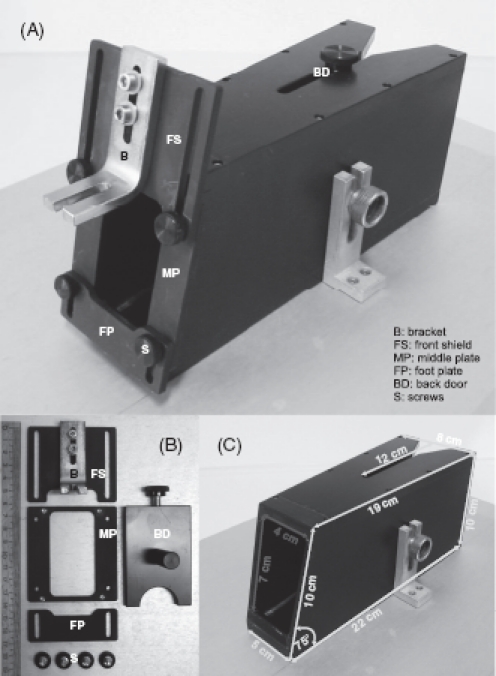
The restrainer. (A) Complete view with assembled parts. (B) Disassembled parts (ruler shows centimeters). (C) Measures in yellow are outer diameter, in red inner diameter. Floor and ceiling of the box are parallel.

*Restrainer*. Head-fixation should be done using a restrainer of some sort. We strongly discourage head-fixating a rodent in open space (without covering the body) as this is experienced by the animal as an exposed and threatening position and thus generates stress and discomfort. The restrainer we use is made from black plastic, the front plate is made from anodized aluminum dyed black (Welsh 1998). An alternative is to put the animal in a black tissue bag that can be tied to enclose the body of the animal snugly and to prevent extension of the forepaw (Bermejo et al. 2004). The restrainer must be sufficiently narrow so that the animal cannot turn inside, but wide enough to allow the animal to fit snugly which is something the animals appear to experience as calming. For this purpose the restrainer is of conical shape with the opening at the back end (through which the rat enters) wider than the opening at the front (through which the head is extended during head-fixation and through which the rat leaves). The dimensions of the restrainer as given in Figure 2 are appropriate for rats weighing around 250–400 g. The front shield holding the bracket for head-fixation can be moved in the vertical direction to adjust the height of the head relative to the body. This vertical position is important to allow the rat to assume a comfortable posture and should be optimized for each individual. A well-habituated rat can be head-fixed and then the vertical plate released. The rat will adjust its head at the most comfortable height which then can be marked and used in further sessions. In addition, the front end has a foot plate that can be adjusted in height to allow a comfortable rest for the forepaws but prevents the extension of the limb towards the snout. This foot plate can be removed to allow access to levers in front of the animal. At the back end a door can be slid in and fixed using a screw. This back door has an opening to accommodate the tail of the rat.

**Figure 3:**
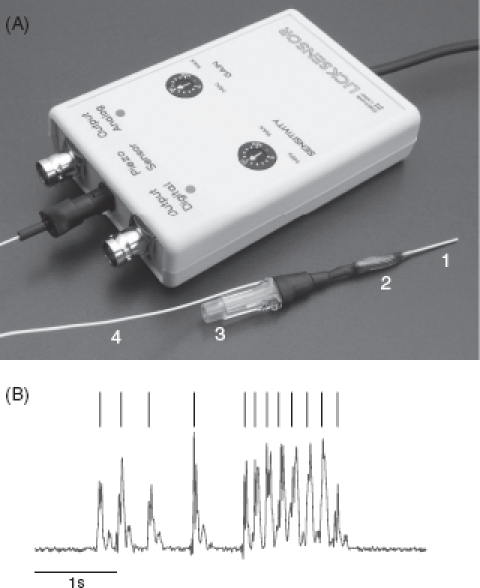
Licking spout. (A) Spout with electronic equipment (“lick sensor”) containing amplifier, high pass filter, and threshold detector. The spout consists of (1) the metallic part of a venous catheter, (2) a piezo element (green) glued to the spout using epoxy glue, the connector to the water tube (3), and the cable carrying the piezo output (4). (B) Analog piezo output (red, arbitrary units) and licking events detected by a simple threshold algorithm (black).

*The licking spout*. The spout and its components are shown in Figure 3. The spout is usually made from a plastic venous catheter. We also use steel catheters, but have found that they generate large licking artifacts in electrophysiological recordings. The spout is coupled via a magnetic valve (Med Associates, St Albans, VT, USA) to a water reservoir located at a level about 1 m higher than the spout.

Thus, the opening time of the magnetic valve can be used to titrate exactly the amount of water emerging by gravitational force at the tip of the spout. A miniature piezo element is glued to the catheter with epoxy glue. Its output voltage is amplified, high pass filtered, and digitized by a comparator circuit with adjustable threshold for detection of a lick.

**Figure 4:**
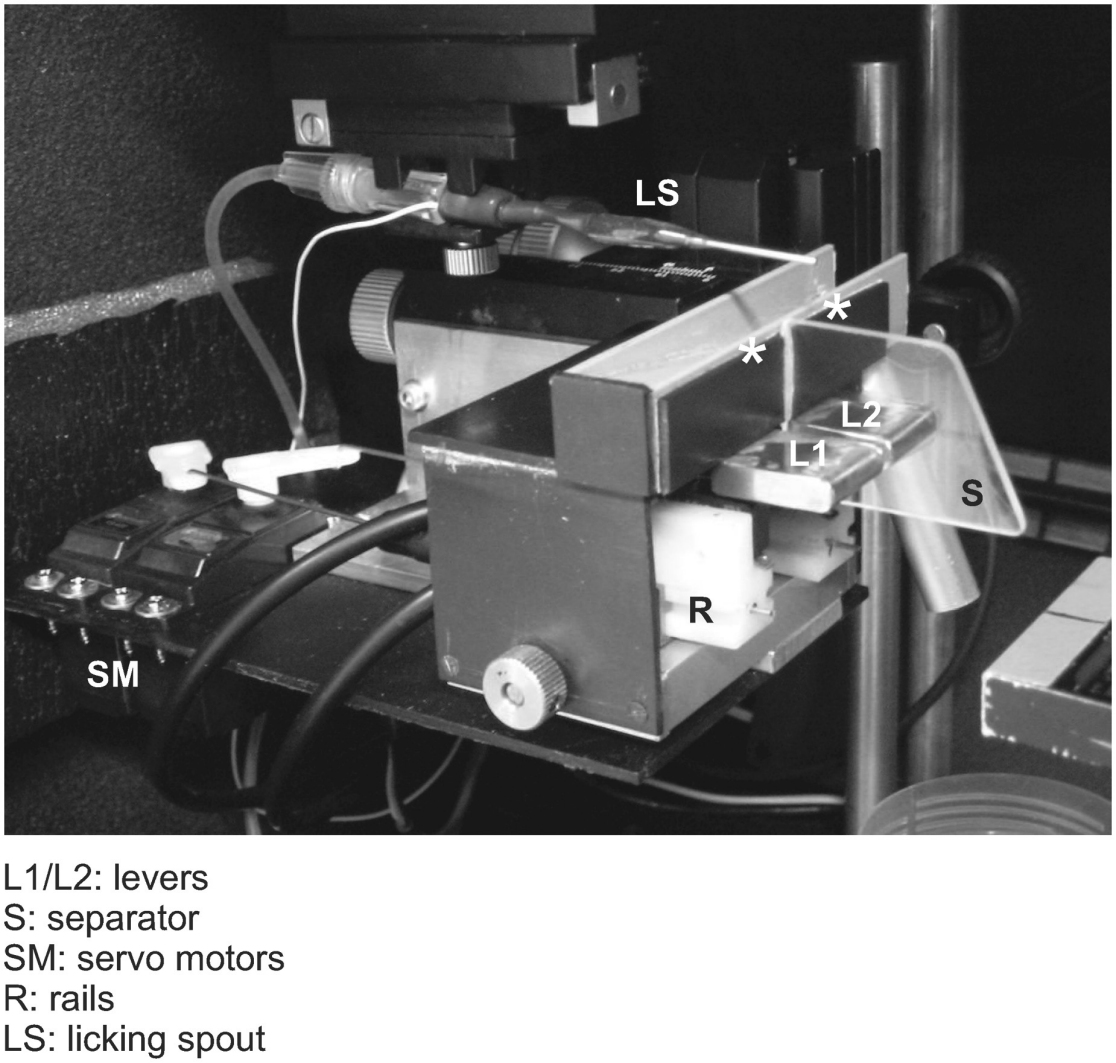
Operant levers. The two levers are separated by a Plexiglas separator to prevent pressing the lever with the wrong paw. Servo motors are used to retract the levers out of the reach of the animals. The licking spout is mounted as well. Asterisks mark the sites where the rats typically like to place their forepaws.

*Levers*. To separate consummatory response (licking) from indicator response, we have introduced lever presses as indicator response. Figure 4 demonstrates two custom-made levers attached to micro-switches which are covered by a plastic box to protect them from water that drops from the drinking spout. The contact travel of the levers is 1 mm and switching contact is signaled by a distinct click that can be heard and sensed at the paw. A Plexiglas separator prevents presses with the contralateral paw. Each lever is mounted on a guide rail and attached to servo motors (Conrad Electronic, Stuttgart, Germany). This allows the retraction of each lever behind a plastic barrier. The possibility to move the lever outside the reach of the animal is an important element of behavioral training. The micro-switches are connected to a custom-made electronic device that generates TTL pulses when the lever is pressed.

*Whisker contact detector*. The whisker contact detector is based on a magnetic cartridge used in record players (Ortofon, Nakskov, Denmark). The stylus carries a glass rod glued to it by a drop of epoxy. The cartridge's output voltage is amplified, high-pass filtered, and digitized by a comparator circuit with adjustable threshold.

*Whisker tracking*. The whisker is elongated by slipping on a light polyimide tube (diameter: 250 μm; length: 2–2.5 cm; weight: ∼0.7 mg) such that it covers the hair 3–5 mm from its base to its free end. It is tracked by shining a two-dimensional laser beam onto a linear CCD array, and tracking the shadow of the tube (Metra-Light, San Mateo, CA, USA) as described in Bermejo et al. (1998). A custom-made version of this device that is able to track whiskers without the tube was described recently (Wolfe et al. 2008).

### Ergonomics under head-fixation

**Figure 5:**
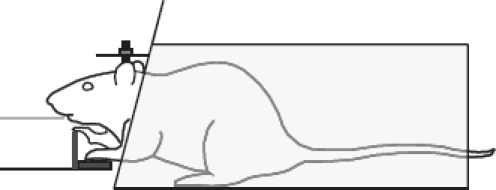
Ergonomics under head-fixation. Schematic of a head-restrained rat inside the restrainer and with position of water spout (blue), paw rest (gray), and lever (red). The relative size of rat and objects and their distance are to scale. Note the slight elevation of the rostral body part and the paws with respect to the floor of the restrainer. The vertical position of the tip of the spout is on par with the rat's lower lip (rostral distance 3–5 mm, see text). The resting position of the paw is elevated and the rat presses the lever using a downward movement that is far from fully extending the forelimb.

The restrainer described above allows the animals to assume a typical posture in which they rest on their flexed hindlimbs with the rostral portion of the body slightly elevated. In the head-fixed situation it is thus comfortable for the animals to rest the forepaw in an elevated position—either on the lower front plate (cf., Figure 2), or if this part is dismantled (e.g., to allow lever presses), on the rim above the levers (Figure 5 gray bar; cf., asterisks in Figure 4). Instruments and manipulanda have to be arranged such that the animal can perform the trained movements in a convenient and ergonomic way (Figure 5). The optimal position for the licking spout is directly in front of the lower lip at a distance of 3–5 mm (blue in Figure 5). By lowering the jaw and extending the tongue the rat can lick in a comfortable way. Positions closer to the lower jaw are unfavorable. They enable the rat to touch the spout with teeth and lip because the lower jaw is movable in the rostral direction. To reduce impulsive licking the animal's cost of licking can be increased by retracting the spout to a distance of 6–7 mm from the lower lip. At this distance the effort the rat has to make to successfully lick at the spout is significantly increased (caution has to be exercised as larger distances preclude licking altogether). Levers are located directly beneath the resting position of the paws (Figure 5 levers red, resting position on gray bar, cf., asterisks in Figure 4). Thus the lever can be pressed by a small and convenient downward movement which does not fully extend the elbow. Figure 5 shows the typical position of the paw while at rest and during a lever press.

### Water control

For the trainer of rodents using water control, it is important to realize that physiological principles governing water homeostasis of these animals are quite different from those in humans. The daily water intake is highly variable and depends on multiple physiological and environmental factors including body weight and age. In adult rats it is on average 20–30 ml (ranging between 5 and 80 ml), and adult mice drink between 3 and 7 ml a day. Compared to humans, the concentrating power of the rat and mouse kidney is much higher (urine osmolarity in mosmol/kg after 24 h total deprivation: man ∼1100; rat ∼1800; mouse 1950–3000 depending on strain) (Ramsay et al. 1977; Rolls et al. 1980; Esther et al. 1996). Mice are exquisitely adapted to life in semiarid environments and can reach levels of urine osmolarity typically seen in desert animals. Rats can survive complete water deprivation for more than 12 days, during which they lose 40–50% of body weight (e.g., Jakubczak 1970), while mice under specific conditions (humidity, water content of food, and rapidity of adaptation) can even attain stable body weight (Haines and Schmidt-Nielsen 1967; Castel and Abraham 1972). These remarkable abilities must be compared to humans who die within 3–5 days after losing 15–20% of body weight. In rats, a deprivation period of 48 h (corresponding to 10–13% reduction of body weight) has been reported to introduce neither strong physiological stress nor behavioral abnormalities (Baetjer 1973). If access to water is restricted to a non-zero constant level, food intake and body weight of rats and mice are reduced and assume a constant level depending on the grade of restriction. Under normal laboratory conditions, rats tolerate 61 % of normal drinking volume, which reduces their weight to a stable level around 90%, while body weight does not stabilize, slipping to critical levels if drinking volume is below 36% (Collier and Dnarr 1966). Again, mice fare better. In the extreme, wild mice reach a stable body weight even when challenged with 12.5% of normal drinking volume provided that the adaptation is slow (Haines et al. 1973). It is interesting to note that rats (and likely mice as well) generally cope much better with restriction of water than with restriction of food (Barker and Adolph 1953; Treichler and Hall 1962)—in sharp contrast to humans who show the reverse order of susceptibilities.

It is important to emphasize that for the present purpose of behavioral training, animals do not need to be *deprived of water* for longer than ∼ 12 h. It rather suffices that the access to water is *controlled* such that they get *sufficient* volume of water *exclusively during* the daily training sessions. It is very important to acquaint the animals to this rule early on. The best time to start water control is the first day of habituation to head-fixation. In this early phase of training it is very easy to fine-tune the volume of water according to the needs and abilities of the animals (i.e., a volume of 1 ml or more can be given for a successful action, e.g., walking through the restrainer etc.). The association with reward gives the sessions a clear appetitive character which is very helpful for habituation to potentially stressful situations and learning the task later on (see next section). During the entire behavioral training, the trainer must decide from day to day when to enter the next training step and must finely adjust the volume of reward received after each successful trial such that satiation can be reached given the performance of the animal. It is always preferable to arrange for an additional training session at the end of a day or to go back one training step to make sure that the rat reaches satiation rather than giving water for free.

**Figure 6:**
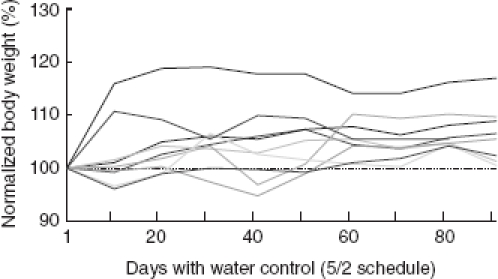
Normalized body weight of nine female SD rats under 5/2 water control schedule (i.e., access of water was controlled for 5 days followed by 2 days of free access). Weights are plotted as the means taken from a 2 week period (i.e., 10 water control days) showing the trend with which the rats gained weight under water control. Note that on days with free access to water body weight was not measured—these days were thus not included on the abscissa reading. The weight on the first day is taken as 100% (broken line). In the represented period of time these rats were first habituated to head-fixation and then trained on a Yes/No discrimination task. Rats gained up to 20% weight during the time of 5/2 water control. This is in the range of weight gain shown by female rats with free access to water.

If these rules are followed, rats on water control drink far above the physiological limits mentioned above—typically they reach complete satiation. Our general strategy is to maintain or slightly increase the animals' body weight during the period of behavioral training (Figure 6). This is the regime that optimally combines well-being of the animal with the high motivation needed for successful work on the task. As a general rule, water is controlled 5 days a week and free access to water is available on weekends (5/2 schedule). In view of the good adaptability of rodents to periods of water deprivation as detailed above, we strongly discourage the use of alternating schedules (e.g., 1 day water control, 1 day free access) or daily periods of free access (e.g., 1 h free access daily). Rats on such schedules will not develop motivation to work for water. In case an animal loses weight over several days (despite interference of the trainer to facilitate the task, see next paragraph), it is supplemented with additional water. Using the 5/2 schedule with the mentioned precautions, we never observed severe dehydration or other health problems related to water control. Moreover, the animals do not show behavioral anomalies, can be easily handled, explore their environment, and groom sufficiently to keep their fur clean.

### Habituation to head-fixation

Our training approach takes advantage of graded exposure techniques. These techniques are based on human behavioral modification which shows excellent results on patients suffering from phobias. Similar techniques have been used with primates (Laule et al. 2003). The approach is based on the pairing of gradually presented anxiety-provoking stimuli with anxiety-competing responses—liquid reward in the case of our rats. The effectiveness of this approach has been shown in conditioning studies in rats (Wilson and Davison 1971). In the context of our work, the approach involves graded repetitive presentations of individual aspects of the final testing situation. Each aspect is paired with reward over multiple days until the animal retrieves the water reward without hesitation. Firstly, the rat is exposed to the working bench with free water reward offered via a syringe. Next, the animal is placed inside the restrainer and water reward is offered as soon as the animal exits the box. Very quickly the rat learns to run through the tunnel in order to obtain water. Fixation is gradually introduced by gently holding the head post (1–2 s) and simultaneously offering water reward as the rat exits the box. The time the head post is hand-held is slowly extended over a number of days, water reward is provided intermittently, and the amount of water provided on each trial is reduced (from about 1ml to 0.1–0.2 ml). In our experience the head post can safely be screwed in place once the animals accept being hand-held at the post for about 30 s. At this point the head-fixed rat can be placed in front of a spout through which water reward is made available drop by drop at a fixed interval schedule of about 1 s. Session length is solely determined by the rat's willingness to retrieve the water reward. This has to be taken literally: a rat that refuses to lick off water is either satiated or stressed—both conditions demand immediate release in order not to endanger the success of behavioral training. Multiple daily sessions may be performed and session duration will eventually progress to about 5 min.

The adaptation to all circumstances of the experiment is done using small steps and by using systematic habituation or de-sensitization. A general rule for the training of the animal is that the next step is only initiated after the last one has been mastered without fear, stress, and struggling. Another important rule is that the steps and manipulations always follow the same order, happen in the same context, and are guided by the same trainer. Successfully completed steps are systematically rewarded with water. Here it is important to note that the aims of animal welfare and science go hand in hand. The more happy and healthy the animal, the more stable the performance, and the better the psychophysical result will be. There is *absolutely* no way to get viable psychophysical results from a stressed or dehydrated animal!

### Conditioned movement

Whisker movement can be conditioned by rewarding the touch of a real object (Hentschke et al. 2006) or by rewarding a whisker movement to a certain point in space (Bermejo et al. 2004). The first stage after habituation of head-fixation is the presentation of a drop of water using a fixed interval of 1 s. Once the rat licks regularly for the water reward, the reward is made contingent on whisker contact with an object that is attached to a contact detector (see description of equipment) and is positioned such that the movement of the whisker due to an opening of the mouth for licking suffices to generate a touch (or reaching the position that triggers reward). During these sessions the object or the reward triggering whisker position is moved slightly forward, so that small whisker movements are required to reach the respective position or to generate the touch. The presence of an object will facilitate learning because it cues the reward by a tactile signal. Once the rat actively moves the whisker, the position required to reach for a reward is incrementally moved rostrally, farther away from the animal to require larger whisking amplitudes. The rats typically tend to hold their whisker close to the object or the trigger position in order to avoid large amplitude whisks. Moving the required position farther forward will ultimately discourage this strategy because it requires force and energy to hold the whisker in a protracted position. At this point the animal will change strategies and generate large amplitude whisks starting from the resting position. Once this behavior is well established, the trigger position can be shifted backwards again without the rat resorting to its previous strategy of holding the whisker in a protracted position between trials. Thus, this paradigm also allows for the implementation of interspersed “passive” contacts, that is, the dynamic movement of the object against the whisker at rest (Hentschke et al. 2006).

Paw movements aimed at pulling a handle or reaching for food have been conditioned under head-fixation as well (Heck et al. 2007; Isomura et al. 2009). Conditioned indicator responses (licks and lever presses) are described in the sections on psychophysics below. Online control of the hardware and analysis of the animals' behavior during this and the following experimental paradigms (see below) were implemented via in-house software written in LabView-Realtime and standard multi-purpose AD/DA boards (National Instruments, Austin, TX, USA).

### Go/NoGo psychophysics

The Go/NoGo task is one of the standard paradigms of animal psychology (Blough and Blough 1977). In its simplest form, the Go/NoGo task requires the animal to emit an operantly conditioned response in the presence of one kind of stimuli (CS+) and not to emit this response in the presence of another kind of stimuli (CS−).

*Detection*. To test the psychometric curve for detection of brief whisker deflections, we defined the occurrence of a brief whisker deflection as CS+ and its absence as CS−. After habituation to head-fixation, the animals are first put on an autoshaping-like procedure (Brown and Jenkins 1968). Whiskers are deflected for 1 s with a suprathreshold sinusoidal vibration (> 300 μm amplitude, >89 mm/s peak velocity at 5 mm distance from the skin) at irregular intervals (5 s ± 1.25 s, flat probability distribution) (Stüttgen et al. 2006). Immediately following stimulus offset, a droplet of water becomes available at the water spout (delay conditioning) for the animal to lick off. Importantly, water is delivered prior to stimulus offset if the animal emits a lick response during stimulus presentation. In our experience, rats start emitting lick responses to the whisker deflections within a few sessions and show stable performance after 1 week. At this stage, water is only delivered after the rat licks at the spout, turning the procedure into an operant conditioning paradigm with the whisker deflection serving as CS+. During the next few weeks, stimulus intensity is gradually lowered and stimulus duration is shortened, while at the same time performance is maintained by the presentation of suprathreshold stimuli in addition to the progressively weaker deflections, until performance does not improve anymore over several sessions. The animal now responds to suprathreshold deflections with extremely short response latencies, averaging about 250 ms, with latencies usually being somewhat longer for weaker (but still detectable) stimuli. Usually, we count licks as responses only when emitted during the first 600 ms following stimulus onset (“window of opportunity”), but this interval depends on the details of the task. Both longer and shorter intervals are feasible. Detection of salient suprathreshold stimuli can be trained within a few sessions after habituation to head-fixation. Stable thresholds in response to a range of stimuli covering the perceptual range, needed to obtain viable psychometric data, are typically reached after about 4 weeks of training.

*Discrimination*. Discrimination training commences in the same way as detection training—the animal is trained to associate a tactile stimulus with water reward. We have used a 90 Hz whisker vibration stimulus with displacement amplitude 11.3° and duration 1 s (Gerdjikov et al. 2010). As with detection, reward is provided automatically at stimulus offset (regardless of the animal's behavior) until the animals show a stable response. Then reward is made contingent on the rat emitting an instrumental lick to provide a clear indicator response. The inter-trial intervals used are longer than those for detection tasks (15–25 s) with 10 s time-out to discourage impulsive licking (see below). Next, the stimulus duration is extended to 5 s and a window of opportunity is introduced which starts with stimulus onset and lasts for 2 s. As before, the window of opportunity is the period in which a response is counted and, if correct, leads to a reward. The onset of the window of opportunity is then gradually shifted until it starts 500 ms before stimulus offset (i.e., 4.5 s after stimulus onset). The purpose of the shift is to reduce impulsive licking after stimulus onset and to make the animal focus on the discriminative stimulus parameters before taking a decision. Shifting should proceed very slowly (1–2 weeks) and in small steps—100–200 ms per individual session. The animal should be immediately removed from the apparatus if it ceases to respond—we have found that three omitted stimuli in a row is a good rule of thumb to use for removing the animal from the apparatus. Once responses are stable, an easy discrimination is introduced, in our case by interspersing the 90 Hz stimuli (CS+) with 15 Hz stimuli (CS−) of the same duration. However, to avoid frustration at least 50% of the trials should remain rewarded (CS+). Responding to the CS− is discouraged by switching a light on for 5 s if a lick is emitted during the window of opportunity. The advantage of the light feedback is that depending on intensity and duration it can be adjusted from a neutral cue to a mildly aversive one. Even when neutral, it quickly conditions as a reward omission cue and the rats will be motivated to avoid it. Other unconditioned punishments like gentle air puffs (O'Connor et al. 2010) or time-outs are feasible as well. As a general rule, the intensity of punitive measures under head-fixation has to be finely adjusted to generate very mild aversive effects. This has to be done individually for each animal. Overly aversive stimuli under head-fixation are discouraged as they entail the risk that the animals stop working altogether. Once the rats grasp this task, a range of non-rewarded CS− stimuli (15–75 Hz in our case) are introduced. Psychophysical testing is conducted using the method of constant stimuli. Stimuli are always presented in blocks of ten. Stimulus order is chosen randomly within each block and across blocks. A single block consists of five rewarded stimuli at 90 Hz (at full or reduced amplitudes, respectively) and five non-rewarded stimuli. The training of a simple discrimination takes about 4 weeks after habituation to head-fixation. Working the animals down to a stable threshold takes another 4 weeks.

*Impulsivity and motivation*. Whenever possible, psychophysical tests using the Go/NoGo paradigm should include measures that monitor the *motivation* of the subject (“does each and every stimulus perceived as CS+ lead to a response?”) and its *impulsivity* (“is a response to a CS− due to internal, non-sensory drive or is it under stimulus control?”). In the detection task, we, therefore, used an array of stimuli containing not only deflections close to threshold, but in addition “reference stimuli” (strong suprathreshold deflections) and “catch trials”. Reference stimuli serve to constantly monitor motivation of the animal over the session. Clear suprathreshold stimuli usually should yield correct GO responses close to 100% in Go/NoGo detection tasks, if the motivation of the animal is high. Catch trials, on the other hand, occur with equal frequency as the single instance of a CS+, but no stimulus is actually presented. Thus, lick responses during an equally long time period are registered as a measure of the “false-alarm rate”. The reason for this is that occasionally rats tend to emit licks randomly during the session to maximize the chance of responding to non-detectable stimuli. The response rate to the null stimulus during catch trials yields a measure of response due to a random-licking strategy. In general, in detection tasks, we aim for a low but measureable false-alarm rate on the order of 10–20%. If lower, one runs the risk of overestimating detection threshold with highly conservative subjects (Swets 1961). If higher, the actual measurement range of the psychometric function is progressively decreased.

Discrimination tasks using the Go/NoGo paradigm are more difficult to monitor because presentation of reference and catch stimuli are typically not feasible. In these cases it is of particular importance to discourage impulsive licking. Toward this aim long inter-trial intervals (up to 30 s) are effective. Often they can be reduced again after the animal has stopped impulsive licking. A further effective measure to avert impulsive licking is to introduce a timeout if the animal emits a lick in a no-lick window prior to stimulus presentation. The time-out clock is reset with every subsequent lick, so that a stimulus never follows a lick by less than the duration of the time-out. With these measures in place, a negligible number of responses is seen during the inter-trial interval in well-trained rats.

Impulsivity and motivation in a Go/NoGo discrimination task can be measured using the percentage of correct responses—but only if the following requirements are met. First of all, the stimulus array must contain a pair of discriminanda that is easily discriminable. Second, the optimal criterion to discriminate all CS− from CS+ must as well be adequate to separate the two easily discriminable stimuli. These two requirements are met if, for example, the parametric range covered by the stimuli is wide and the division line between CS− and CS+ stimuli divides the whole stimulus ensemble into two symmetric groups. This arrangement guarantees that the criterion applied to discriminate the two groups of stimuli is optimal as well to discriminate the two most dissimilar stimuli. In this case absence of impulsivity and highest motivation should lead to response rates close to 0 and 100%, respectively, for the two most dissimilar stimuli. The situation is different if the dividing line between the groups of CS− and CS+ is asymmetric with respect to the parametric range spanned. Let us assume the stimulus ensemble is divided into just one CS+ and many CS−. Then, perfect discrimination between the CS+ and the most similar CS− is likely to be impossible and the response rate for the CS+ will be lower than 100% for sensory/perceptual reasons (cf., Gerdjikov et al. 2010). Consequently, response rates cannot easily be interpreted as reflecting the level of motivation in this case. A similar argument can be put forward for the monitoring of impulsivity in the inverted case where the stimulus ensemble is composed of only one CS− but many CS+.

For all Go/NoGo paradigms, one has to be careful not to frustrate the animal by presenting too many near-threshold stimuli during the course of an experimental session. We usually present 6–12 stimulus types (including reference and catch stimuli), and we compose the stimulus set such that the animals are able to retrieve reinforcement in a minimum of 50% of trials.

*Limitations of the Go/NoGo approach*. Despite its demonstrated usefulness for the assessment of psychometric functions, the Go/NoGo task has important limitations. First of all, true signal-detection responses are confounded with random guessing when the animals emit random licks. This can, however, to some degree be controlled by including catch trials and discouraging random responses as detailed above. Secondly, true signal misses are confounded with lack of motivation (i.e., satiation or frustration). As a control, reference stimuli can be employed. Thirdly, especially when used as a detection task, the asymmetric contingencies can become problematic: hits but not correct rejections are rewarded, while false alarms but not misses are punished. This way, the animal lacks feedback about both misses and correct rejections. One might argue that rewarding correct rejections and punishing misses may solve this problem, but this procedure may confuse the animal in the case of near-threshold stimuli, where it sometimes receives reward (for a correct rejection) and sometimes punishment (for a miss) when its internal state (no signal present) is actually the same. Below, we discuss a classic psychophysical paradigm which alleviates these problems: the Yes/No task.

### Yes/No psychophysics (Y/N)

In Y/N paradigms the response to one manipulandum is reinforced if a specified condition is present (“yes” response), while a response to another manipulandum is rewarded if the condition is absent (“no” response) (Blackwell 1952; Green and Swets 1966; Blough and Blough 1977). To test discrimination, Y/N procedures typically consist of the presentation of one stimulus per trial upon which the subject commits to one of two possible responses depending on the perceived value of a certain stimulus parameter. The Y/N procedure has been traditionally separated from the so-called forced choice tasks (FC) in which a number of *n* stimuli (often *n* = 2) are presented synchronously or in seamless sequence and the subject must choose from *n* manipulanda to indicate its decision. Unfortunately, the terminology of psychophysical tasks is a bit tangled, and thus warrants a note of clarification: the categorical distinction between Y/N and FC is not the absence of a forced choice—indeed, the Y/N paradigm does contain a forced choice component (i.e., the two manipulanda), and thus, is sometimes referred to as Single Interval Forced Choice task. Rather, the distinction between the two paradigms is the different cognitive mechanisms that are assumed to give rise to the subject's decision. In Y/N tasks the presented discriminandum must be compared to the content of long-term memory, while in FC tasks all stimuli are represented in the sensory neural system at the same time (or in seamless sequence), such that recall of stimulus characteristics stored in memory is not necessary to solve the task. In their comprehensive review of methods in animal psychophysics, Blough and Blough (1977, pp. 518–519) argue that Y/N tasks are a good alternative if FC procedures turn out to be too difficult to learn for animals.

We have trained rats to associate two temporal frequencies of pulsatile whisker deflections to two levers installed directly in front of either of its paws (Figures 4 and 5). The head-fixed rat was free to move its front paws to be able to handle the levers. The levers are arranged side by side and movable on rails via servo motors such that they can be presented to the rat or withdrawn behind a plastic case. Between the levers there is a separator that enables the rat to press each lever only with the respective paw. Arbitrary paw movements (grasping the water spout or stimulator, grooming, etc.) can be blocked by introducing plastic plates between the rat's face and the lever space.

**Figure 8:**
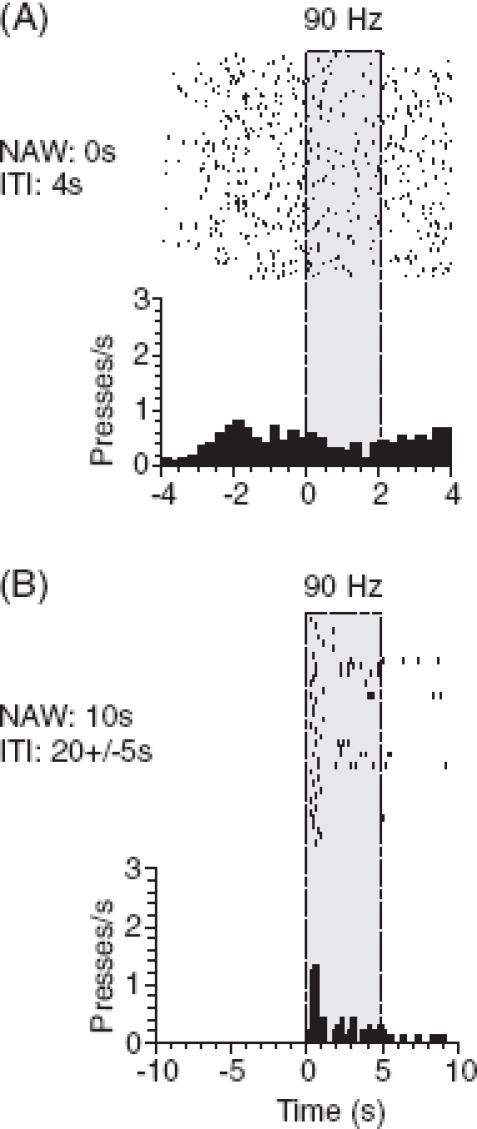
Discrimination performance using a Yes/No psychophysical paradigm. (A) Schematic of the task (time runs on a horizontal axis but is not to scale). Reward is given for correct responses emitted within the window of opportunity (gray). A light signals [see description in the section about Go/NoGo detection paradigm] signal false responses and absence of reward. The light signal is not given if no response is emitted (not shown). (B) Raster displays and peri-event histograms for presentation of the two conditioned stimuli (CS1 and CS2). The duration of the stimuli (repetitive whisker deflection at different inter-pulse frequencies) is marked by the brackets. The NAW preceding the stimulus onset (broken line) was 6 s in this experiment.

We have successfully trained head-fixed rats to discriminate two vibrotactile stimuli consisting of pulses at a frequency of 10 and 90 Hz at a rate of correct responses of over 80% (Figure 8). In our hands the time to train the animals to perform this simple discrimination is ∼10 weeks after habituation to head-fixation.

**Figure 7:**
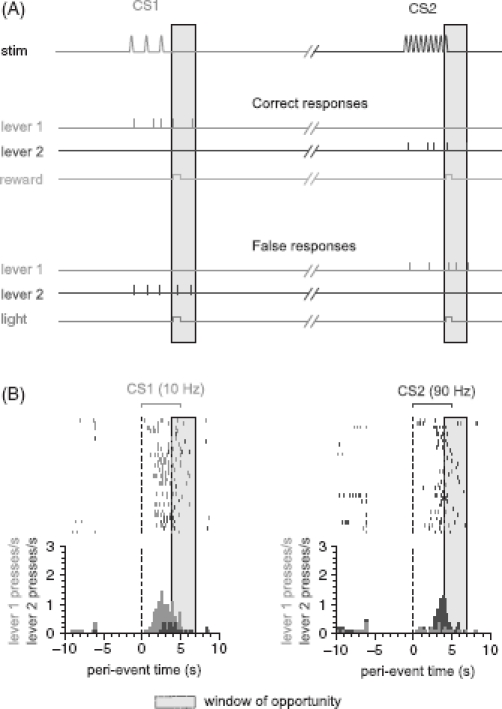
Impulsive responses. (A) Detection task using lever presses as indicator response. Raster display and peri-event histogram of lever presses before and during stimulus presentation (gray) with inter-trial intervals (ITI) of 4 s. (B) Later during training of the same rat impulsive responding was greatly reduced by increasing ITI to an average of 20 s and introducing a no-activity window (NAW) preceding the stimulus by 10 s.

*Stimulus-reward association*. The first step after the habituation to head-fixation is the association of stimuli with reward using classical conditioning. This association is a major building block and helps to fill in necessary additional steps later on (Dickinson and Dawson 1987). All conditioned stimuli (CS) are presented to the animal (in our case: different frequencies of vibrotactile whisker deflections) followed by a fixed automatic water reward. Impulsive lever pressing is reduced by long inter-trial intervals and a no-response window as described above for Go/NoGo paradigms (Figure 7). After very few sessions the animals show licks toward the end of stimulus presentation.

*Operant conditioning under stimulus control*. As a next step the animals are trained to respond to a 2 s vibrotactile stimulus using a lever press. For each session another discriminandum and its associated lever (left or right) are used. The unused lever is retracted, and thus, is out of reach of the animal. In the beginning the rats are gently prompted to press the lever by placing their paws onto the lever and assist lever presses by using a hand-held cotton swap. However, prompting should be used with caution to avoid the conditioning of the response to the action of the trainer rather than to the stimulus. To receive a drop of water, the animals must respond during a defined time window (window of opportunity), which is identical to stimulus duration. Catch trials are presented as well to monitor impulsive pressing. Once impulsive presses are absent and responses to the stimuli are stable, the duration of the stimulus is extended stepwise to 5 s and the window of opportunity is shifted step-wise (see Go/NoGo discrimination) toward the end of the stimulus.

*Stimulus discrimination*. Once the rats are able to flexibly switch between the levers from session to session, the discriminanda are presented in a randomized sequence. Figure 8 depicts the final psy-chophysical paradigm and one example session for a representative animal in the final stage of training with a rate of correct responses of 80%.

*Limitations of the Y/N task*. As in Go/NoGo paradigms, responses can partly or entirely evade stimulus control. In the framework of Y/N tasks one reason for evasion of stimulus control is known as response bias. A bias exists if the subject exercises a stereotyped response pattern that is partly or totally independent of the stimulus presentation. In severe cases rats only respond to one side alone and completely ignore the other one (alternating biases that are sometimes observed using freely running rodents were not observed in the head-fixed rats). To avoid biases, rats are subjected to whole sessions in which only the stimulus associated with their non-preferred lever is presented (with both levers in place and reachable). Algorithms exist which automatically change the probability of stimuli depending on response biases (Knutsen et al. 2006). It is important to note that bias correction necessarily introduces cues contained in reward and response history, and thus, may compromise stimulus control. Therefore, bias correction serves its purpose best in the training phase. The final measurement of psychometric curves should be done without bias corrections.

## Discussion

In this paper we present an array of behavioral techniques suited for the investigation of cognitive, perceptual, and motor behavior in head-fixed rodents. Our focus was on the study of (active) perception in the rodent's whisker-based tactile system. However, we expect the head-fixed behaving rat preparation to be beneficial in many fields of systems neuroscience. Traditional neurobiological techniques have been successfully combined with behavioral observation of spontaneous or conditioned movements. Lesions of motor cortex (Gao et al. 2001), extracellular recordings (Hentschke et al. 2006; Stüttgen and Schwarz 2008; Khatri et al. 2009) as well as microstimulation or juxtacellular stimulation in sensorimotor cortex have been performed (Haiss and Schwarz 2005; Butovas and Schwarz 2007; Houweling and Brecht 2008). These approaches put the rodent preparation on a par with long-established methods in awake behaving monkeys (Evarts 1968a). The benefits and prospects of the rodent preparation, however, emerge from the relative ease with which methods commonly used on the cellular and molecular level of investigation can be accommodated. Intracellular recording techniques, most notably whole cell patch clamp techniques, have been made available for the use in awake head-fixed rodents (Brecht et al. 2004; Crochet and Petersen 2006; Melzer et al. 2006; Harvey et al. 2009). Recently parallel recordings from two neurons have been achieved (Poulet and Petersen 2008; Gentet et al. 2010). Furthermore, traditional cellular techniques such as imaging with single cell resolution, commonly used in *in vitro* preparations to date, are being adapted for the use in awake head-fixed rodents. The bulk loading technique of calcium dyes developed in the anesthetized rodent (Stosiek et al. 2003) has been successfully adapted and used in awake head-fixed animals (Dombeck et al. 2007; Greenberg et al. 2008). This method, however, cannot be used repetitively in the same animal. This problem is sought to be solved by novel genetic calcium indicators transfected by viral carriers in the future (Wallace et al. 2008). A huge, so far largely unexploited, potential lies in the riches of genetically engineered mouse lines. In the first pioneering studies, awake (but untrained) mice have been used successfully to study whisker and licking movements (Crochet and Petersen 2006; Poulet and Petersen 2008; Bryant et al. 2009). On a spherical treadmill they could be trained to run within a virtual visual environment (Dombeck et al. 2007; Harvey et al. 2009). Successful operant conditioning of position discrimination via freely moving whiskers touching a pole has been demonstrated recently, for the first time supporting the notion that mice, like rats, are amenable to complex behavior under head-fixation (O'Connor et al. 2010). The potential of rodent genetic models will become fully available if more of the complex sensorimotor, perceptional, and cognitive tasks are adapted to the training of head-fixed mice.

In summary, awake behaving rodent preparations are on the rise because they promise research on the interface between behavior and its neuronal correlates down to function of single cells, cellular components, and specific molecules. However, complex perceptual and cognitive tasks had not been implemented until recently. What are the problems that have so far impeded the breakthrough of the head-fixed rodent preparation in the neurosciences? In our view there have been two major problems. The first one is immobilization. Compared to primates, rodents have been seen by many researchers as less amenable or not appropriate at all for head-fixation. Second, it is well known that rodents can be trained quite easily to tasks involving whole-body navigational movements (e.g., running, nose-poking) which are at the basis of the most common behavioral tests in freely running rodents. Obviously these movements are incompatible with head-fixation. The present paper presents solutions to overcome both of these problem classes.

### Minimizing stress

Stress and aversiveness are unwanted effects that, at the very least, will lead to prolonged training periods needed to condition the animals even to simple tasks—if they do not prevent learning altogether. In order to minimize stress some studies introduced head-fixation under anesthesia and physiological recording was only started after a short waiting period during which anesthesia was thought to subside (e.g., van Looij et al. 2004). The drawback of this method is that long-lasting effects of anesthesia cannot be excluded as a confound. Furthermore, stress will most likely build up after the animals wake up and will interfere with the neurophysiological and behavioral data assessed in this situation. Parry and McElligott (1993) found that conjoint head-fixation of pairs of rats, body to body, effectively reduced stress. While elegantly using the stress reducing potential of social behavior, this method has obvious limitations for the training of more complex behavioral tasks as the interactions between the animals are difficult to monitor and control.

The procedures detailed in this paper were established to cope with these problems. The systematic habituation procedure detailed here adapts rats within 1–2 weeks to head-fixation with stress and struggling being virtually absent. The most important ploy is to use reward as a systematic tool from the outset. It immediately establishes an appetitive link between the whole head-fixation procedure and reward, and thus avoids aversive associations. If well habituated, rats enter the restrainer by themselves, present their heads for head-fixation, do not show any signs of distress (often not a blink of the eye), accept water under head-fixation, and readily eat sweet drops immediately after release.

Stress minimization is beneficial not only for operant but also for simpler schemes of classical conditioning (Koekkoek et al. 2002). We hold it a mandatory step before training complex perceptual, sensorimotor, or cognitive tasks. Furthermore, behavioral training of the task is shortened considerably and it helps to maintain the stability of head-caps. Animals that are calm under head-fixation exert less mechanical stress at the head implants, and thus are less likely to induce atrophy of the bone around the anchoring screws. When using systematic habituation, problems with head-cap stability are rarely observed. In addition, drop-out rats (i.e., animals that have to be taken off the experiment because they do not adapt to head-fixation) are virtually absent. We are convinced that it is the benefits of stress avoidance using the slow habituation method that has allowed us to set up several psychophysical tasks, some of them of quite complex nature: two Go/NoGo detection tasks (Stüttgen et al. 2006; Stüttgen and Schwarz 2008, 2010), one Go/NoGo discrimination task (Gerdjikov et al. 2010), and one Y/N discrimination task, all described in this paper. We expect that these paradigms can be readily adapted to the investigation of the neural bases of other sensory systems, the motor system, or cognition.

### Conditioning movements applicable under head-fixation

A second limitation of the rodent head-fixed preparation is that—compared to primates where hand and finger movements play a dominant role—the behavioral repertoire of rodents includes many whole-body movements that are incompatible with head-fixation. Rats do use their forepaw, but the range of manipulative movements performed is much more limited than that of primates. Most paw movements are used to bring food or other objects close to the snout for further tactile and olfactory investigation or eating. Manipulative actions that reach into spheres farther distant from their own body are typically performed using whole-body movements in combination with head and whisker movements. Even in lever presses, the classical indicator responses used for rodent training in Skinner boxes, a major movement component is a whole-body movement rather than an isolated extension of the forearm. Likewise, an explorative approach toward objects is not typically done using reaches with the paw but instead employs whole-body movements in concert with head, mouth, and whisker movements. Nevertheless, if the experimental apparatus is purposefully designed to preclude whole-body movements, reaching movements using the paw are generated regularly by freely moving rats and are amenable to instrumental learning (Bures and Bracha 1990; Buitrago et al. 2004). More recently, forepaw movements have been trained successfully in head-fixed rats (Heck et al. 2007; Isomura et al. 2009). In summary, most of the rodent manipulative actions aimed at objects outside their immediate body sphere typically involve whole-body movements. In other words, isolated limb, tongue, or whisker movements, as required under head-fixation, are at least an unusual request for the animals. It, therefore, requires specific attention of the experimenter that the type of movement and the animal's posture are selected and adjusted with care to allow successful training.

The first requirement is that the animal be able to assume a comfortable, natural posture. The restrainer detailed here, the principal geometric outline of which was developed by Welsh (1998), allows the rats to assume a natural posture. They are able to crouch inside the box sitting on their hindlegs, with the rostral part of the body slightly elevated, such that the forepaws rest without restraint on the upper edge of the lower front plate (Figure 5). In addition, it provides a tight enclosure that meets the animal's need for a secure and sheltered environment. The second requirement is that the trained movement can be executed in an ergonomic and convenient way. Special thought has to be given on how to arrange instruments and actuators around the animal to allow for natural-like, ergonomic movements, and thus guarantee successful training (Figure 5).

Obviously, many easy-to-train navigational whole-body movements (e.g., nose pokes, locomotion) will continue to remain the exclusive attraction of freely running preparations. Nevertheless, as shown in this paper, a considerable range of movements like whisking, licking (Bermejo et al. 1998; Hentschke et al. 2006; Jadhav et al. 2009), and forepaw movements (lever press: see results; grasping: Heck et al. 2007) can be successfully conditioned for behavioral paradigms under head-fixation.

### Outlook

With the tools presented in this paper research of the sensorimotor neuronal system can be performed with high stimulus and behavioral control. The use of the head-fixed rodent preparation will allow combined research on quite different levels of observation, for example, the cellular/molecular and the behavioral level. Moreover, the study of cognitive abilities of head-fixed rodents becomes feasible. Like sensorimotor research, many such studies will benefit greatly from spatio-temporally precise stimulus control and minute tracking of motor output, both an eminent characteristic of head-fixed preparations.
